# Investigating the Role of the N-Terminal Loop of PD-1 in Binding Process Between PD-1 and Nivolumab via Molecular Dynamics Simulation

**DOI:** 10.3389/fmolb.2020.574759

**Published:** 2020-09-15

**Authors:** Wenping Liu, Haoyu Jin, Ting Chen, Gangping Zhang, Shengsheng Lai, Guangjian Liu

**Affiliations:** ^1^Guangdong Food and Drug Vocational College, Guangzhou, China; ^2^Clinical Data Center, Guangzhou Women and Children’s Medical Center, Guangzhou Medical University, Guangzhou, China

**Keywords:** PD-1, nivolumab, N-terminal loop, molecular dynamics simulation, two-step model

## Abstract

The blockade of immune checkpoints, such as programmed death receptor 1 (PD-1) and programmed death ligand 1 protein (PD-L1), is a promising therapeutic approach in cancer immunotherapy. Nivolumab, a humanized IgG4 antibody targeting PD-1, was approved by the US Food and Drug Administration for several cancers in 2014. Crystal structures of the nivolumab/PD-1 complex show that the epitope of PD-1 locates at the IgV domain (including the FG and BC loops) and the N-terminal loop. Although the N-terminal loop of PD-1 has been shown to play a dominant role in the complex interface of the static structure, its role in the dynamic binding process has not been illustrated clearly. Here, eight molecular systems were established for nivolumab/PD-1 complex, and long-time molecular dynamics simulations were performed for each. Results showed that the N-terminal loop of PD-1 prefers to bind with nivolumab to stabilize the interface between IgV and nivolumab. Furthermore, the binding of the N-terminal loop with nivolumab induces the rebinding between the IgV domain and nivolumab. Thus, we proposed a two-step binding model for the nivolumab/PD-1 binding, where the interface switches to a high-affinity state with the help of the N-terminal loop. This finding suggests that the N-terminal loop of PD-1 might be a potential target for anti-PD-1 antibody design, which could serve as an important gatekeeper for the anti-PD-1 antibody binding.

## Introduction

Cancer, the leading cause of death worldwide, constitutes a considerable burden to society (Bray et al., 2018; Miller et al., 2019). Cancer immunotherapy, that is, harnessing the immune system to battle tumors, has attained remarkable achievement in cancer treatment. Cancer immunotherapy comprises various treatment approaches, including antitumor monoclonal antibodies, cancer vaccines, and antibodies that block immune inhibitory pathways (Mellman et al., 2011; Couzin-Frankel, 2013). Among these treatments, blockade of immune checkpoints is the most promising approach to activate therapeutic antitumor immunity (Ribas and Wolchok, 2018). The 2018 Nobel Prize in Physiology or Medicine was awarded to James P. Allison and Tasuku Honjo for their pioneering discoveries that led to the development of immune checkpoint inhibitors, which block the inhibitory action of T cell molecules, including programmed death receptor-1 (PD-1) and cytotoxic T lymphocyte-associated protein 4 (CTLA-4) (Pardoll, 2012; Guo, 2018; Zaidi and Jaffee, 2018).

PD-1 is an immune checkpoint receptor of the CD28 family expressed in tumor and immune cells (Keir et al., 2008). It is a 288 amino acid type I transmembrane receptor, and its ectodomain consists of four domains, namely, signal peptide, N-terminal loop, extracellular immunoglobulin variable (IgV) domain, and stalk region. The blockade of the interaction between PD-1 and its ligand programmed death ligand 1 protein (PD-L1) was observed to restore the attenuated immune response and lead to increased antitumor and antiviral activities (Hirano et al., 2005; Zitvogel and Kroemer, 2012; Sharma and Allison, 2015). Several PD-1/PD-L1 pathway inhibitors, including pembrolizumab, nivolumab targeting PD-1 and atezolizumab, durvalumab, and avelumab targeting PD-L1, have been approved by the US Food and Drug Administration (FDA) for the treatment of multiple cancers to date (Callahan et al., 2016; Ivashko and Kolesar, 2016; Leventakos and Mansfield, 2016; Bellmunt et al., 2017; Kim, 2017; Muller et al., 2017; Rittmeyer et al., 2017; Sidaway, 2017; Syed, 2017). Humanized IgG4 antibody nivolumab received the most attention and was approved for the treatment of melanoma, metastatic non-small-cell lung cancer, renal cell carcinoma, and Hodgkin lymphoma in 2014.

**FIGURE 1:**
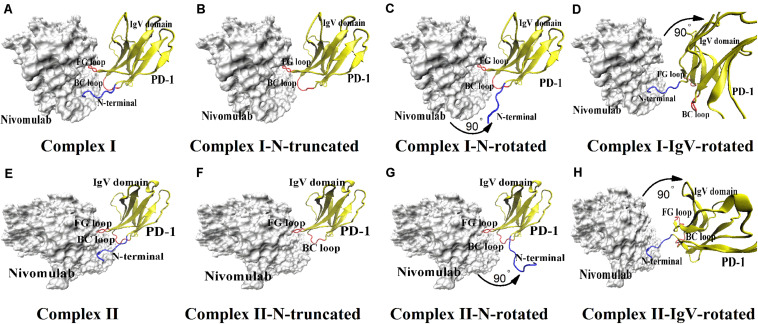
Eight molecular systems of nivolumab/PD-1 complex. **(A)** Complex I. It is downloaded from the PDB with accession code of 5GGR. **(B)** Complex I-N-truncated. It is built through cutting off the N-terminal loop of PD-1 of Complex I. **(C)** Complex I-N-rotated. It is built through the rotation of the N-terminal loop of PD-1 of Complex I. **(D)** Complex I-IgV-rotated. It is built through the rotation of the IgV domain of PD-1 of Complex I. **(E)** Complex II. It is downloaded from the PDB with accession code of 5WT9. **(F)** Complex II-N-truncated. It is built through cutting off the N-terminal loop of PD-1 of Complex II. **(G)** Complex II-N-rotated. It is built through the rotation of the N-terminal loop of PD-1 of complex II. **(H)** Complex II-IgV-rotated. It is built through the rotation of the IgV domain of PD-1 of Complex II. The nivolumab is shown in silver (Surface mode). The N-terminal loop of PD-1 is shown in blue (New Cartoon mode). The FG loop and the BC loop of the IgV domain are shown in red, and the rest parts of the IgV domain are shown in yellow (New Cartoon mode).

Two crystal structures of the nivolumab/PD-1complex have been reported in 2015 and 2016, respectively, providing interface information at the atomic level (Figures 1A,E; Lee et al., 2016; Tan et al., 2017). Three loops of PD-1 provide a flexible platform for nivolumab binding, including the N-terminal loop, the FG loop, and the BC loop. The FG and BC loops locate on the IgV domain. Previous studies showed that the FG loop (i.e., _*PD–*__1_PRO130 and _PD–__1_LYS131) is a binding site for PD-L1 as well as a “hot spot” for several immune checkpoint blockade monoclonal antibodies, such as GY-5 and GY-14 (Zak et al., 2015; Liu and Liu, 2017; Chen et al., 2019a). They clearly suggest a steric clash blockade mechanism of nivolumab. Unexpectedly, the N-terminal loop of PD-1 is far from the interface of PD-1/PD-L1 complex but contributes the majority of hydrogen bonds (H-bonds) to the binding of nivolumab and PD-1. Experiments further proved that the truncation of the N-terminal loop of PD-1 would abolish the nivolumab binding (Tan et al., 2017). Thus, the N-terminal loop of PD-1 plays a dominant role in the complexation of PD-1 and nivolumab. In spite of this, how the N-terminal loop regulates the dynamic binding process has not been answered clearly. Molecular recognition is a dynamic process, and the binding of a ligand to its receptor is regarded not as a single, frozen structure but rather a macromolecule in constant motion (Moroni et al., 2015). Considering the two-site mode (the N-terminal loop and the IgV domain) at the interface of the nivolumab/PD-1 complex, we assumed a two-step binding model, where the N-terminal loop will help switch the binding to a stronger state whether it comes across nivolumab firstly or not.

Molecular dynamics (MD) simulation is well suited for studying the dynamics of proteins (Liu et al., 2018). We have used MD simulations to elucidate conformational selection and induced fit mechanisms in the binding of PD-1 and PD-L1 via MD simulations, and found that the CC’ loop of PD-1 is flexible and switches from an open form to a close one to stabilize the PD-1/PD-L1 complex (Liu et al., 2017). MD simulation is also proven to be a useful tool to detect the “hot-spots” in the complex interface, such as the 6B4/GPIba complex, the 10B12/GPVI complex, the PD-1/PD-L1 complex, and the PD-1/pembrolizumab complex (Fang et al., 2012; Liu et al., 2016; Liu and Liu, 2017).

Therefore, MD simulations were adopted here to investigate the role of the N-terminal loop of PD-1 in the dynamic binding process between PD-1 and nivolumab. Two crystal structures of the nivolumab/PD-1 complex were used to build eight molecular systems with different binding states to mimic the scenarios with or without the N-terminal loop, and the N-terminal loop binds firstly or not (Figure 1). The results show that the N-terminal loop of PD-1 prefers to bind with nivolumab to stabilize the complex interface between the IgV domain (i.e., FG loop and BC loop) and nivolumab. The binding of the N-terminal loop with nivolumab also induces the rebinding between the IgV domain and nivolumab. These findings suggest a two-step binding model, in which the interface of nivolumab/PD-1complex switches to a stronger binding state with the help of the N-terminal loop of PD-1.

## Materials and Methods

### System Setup

Eight nivolumab/PD-1 complex structures were set up for MD simulations (Figure 1). First, two crystal structures of the nivolumab/PD-1 complex with accession codes of 5GGR and 5WT9 were downloaded from the PDB, and were designated as Complex I and Complex II respectively (Figures 1A,E). Both structures include a long N-terminal loop at the complex interface, but in different lengths (_PD–__1_SER27-_PD–__1_ASN33 for complex I, _PD–__1_LEU25-_PD–__1_ASN33 for complex II). Actually, Complex II has an intact N-terminal loop and Complex I only lacks two residues, because the residues before _PD–__1_LEU25 belong to the signal peptide, which will be post-translationally removed and cannot be secreted. Second, the N-terminal loops of Complex I and II were cut off, and the remaining structures were designated as Complex I-N-truncated and Complex II-N-truncated (Figures 1B,F). Third, the N-terminal loop of Complex I and Complex II was rotated backward at the interface with 90° to dissociate from nivolumab, with the IgV domain of PD-1 and nivolumab fixed. These two structures were used to mimic the scenario where the IgV domain of PD-1 binds to nivolumab at the first step and designated as Complex I-N-rotated and Complex II-N-rotated (Figures 1C,G). Finally, the IgV domain of Complex I (_PD–__1_PRO34-_PD–__1_ARG143) and Complex II (_PD–__1_PRO34-_PD–__1_LEU142) was rotated backward at the interface with 90° to dissociate from nivolumab, with the N-terminal loop and nivolumab fixed. These two structures were used to mimic the scenario where the N-terminal loop of PD-1 binds to nivolumab at the first step and designated as Complex I-IgV-rotated and Complex II-IgV-rotated (Figures 1D,H).

The N- and C-terminal residues of each complex were acetylated and amidated, respectively, to mimic the continuation of the protein chains. The missing residues of PD-1 in each complex structure were modeled by the SWISS-MODEL server, with the free PD-1 structures with PDB accession codes 3RRQ and 2M2D as templates (Bordoli et al., 2008; Biasini et al., 2014). The protonation state of each protein residue at neutral pH was determined with the software PROPKA (Bas et al., 2008). Each complex was first solvated with TIP3P water molecules in a rectangular box with walls at least 15Å away from any protein atom. Then, Na^+^ and Cl^–^ ions were added to neutralize the systems at a 150 mM salt concentration. The final system each consists of ∼35,500 water molecules, ∼100 Na^+^ and ∼100 Cl^–^ ions (Supplementary Table S1).

### MD Simulations

VMD 1.9.3 program was used for visualization, modeling, data analysis, and conformation presentation (Humphrey et al., 1996). NAMD 2.11 program with CHARMM36 all-atom force field was used for simulations (Phillips et al., 2005; Best et al., 2012). The cMAP correction for protein backbone, a time step of 2 fs, and a periodic boundary condition were applied in the simulations. The particle mesh Ewald method and a smooth cutoff of 12 Å were employed to calculate the full electrostatic and van der Waals forces.

First, each system was energy-minimized for 5,000 steps with all protein atoms fixed and for another 5,000 steps with all atoms free. Next, each system was heated gradually from 0 to 310 K in 1 ns. Then, equilibrium simulation of 100 ns was performed thrice (named Equ1, Equ2, and Equ3) for each system. A 310 K heat bath was manipulated using the Langevin thermostat, and a 1 atm pressure was controlled using the Langevin piston method during equilibriums.

### Data Analysis

Transient complex formation usually relies on H-bonds (Kar et al., 2012), and they are the dominant linkers at the interface of nivolumab/PD-1 complex (Lee et al., 2016; Tan et al., 2017). Therefore, H-bonds across the interface in simulations were detected using VMD software with in-house scripts. An H-bond was defined if the donor–acceptor distance and bonding angle were smaller than 3.5 Å and 30°, respectively. The survival ratio of an H-bond was defined as the percentage of bond survival time.

The root mean square deviation (RMSD) of heavy atoms was used to illustrate the stability of the structures as well as the conformational changes of the N-terminal loop and IgV domain of PD-1. When analyzing the RMSD for the N-terminal loop and IgV domain of PD-1, the structures of nivolumab were aligned. Buried solvent-accessible surface area (SASA) of each complex, representing the interface area, was calculated using VMD software with handwritten scripts. The interaction energy, mainly including van der Waals and electrostatic energy, was calculated using NAMD Energy plugin (version 1.4) provided in VMD 1.9.3.

## Results

### Interface Analysis of the Nivolumab/PD-1 Complex

To describe the dynamic picture of the interface of the nivolumab/PD-1 complex, two crystal structures of the complex with accession codes 5GGR and 5WT9 were downloaded from the PDB database, and designated as Complex I and Complex II (Figures 1A,E), respectively. They were solvated with TIP3P water molecules in a rectangular box. Equilibrium simulation of 100 ns was performed thrice (named Equ1, Equ2 and Equ3) for each complex after an energy minimization of 10,000 steps. The RMSD of heavy atoms showed that these two complexes had reached a local minimum after 20 ns (Supplementary Figures S1, S2).

**FIGURE 2:**
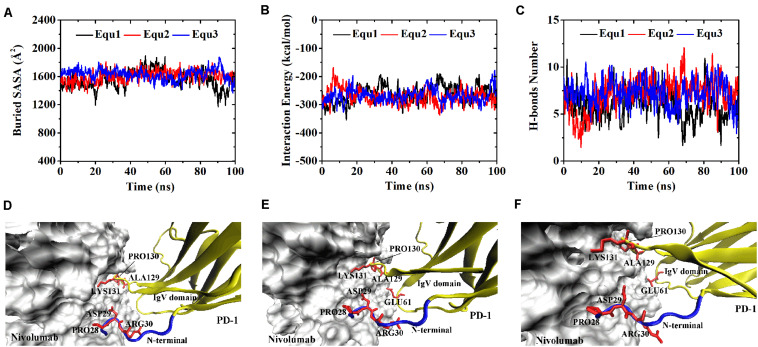
Buried SASA **(A)**, interaction energy **(B)**, and number of H-bonds **(C)** of Complex I in three runs (Equ1, Equ2, and Equ3). **(D–F)** Show the last frame of Complex I in Equ1, Equ2, and Equ3, respectively. The nivolumab is shown in silver (Surface mode). The N-terminal loop of PD-1 is shown in blue and the IgV domain of PD-1 is shown in yellow (New Cartoon mode). The interaction residues of PD-1 are shown in red (Licorice mode).

**FIGURE 3:**
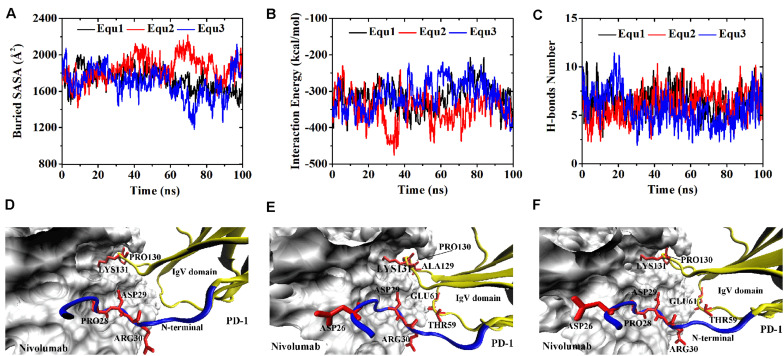
Buried SASA **(A)**, interaction energy **(B)**, and number of H-bonds **(C)** of Complex II in three runs (Equ1, Equ2, and Equ3). **(D–F)** Show the last frame of Complex II in Equ1, Equ2, and Equ3, respectively. The nivolumab is shown in silver (Surface mode). The N-terminal loop of PD-1 is shown in blue and the IgV domain of PD-1 is shown in yellow (New Cartoon mode). The interaction residues of PD-1 are shown in red (Licorice mode).

**FIGURE 4:**
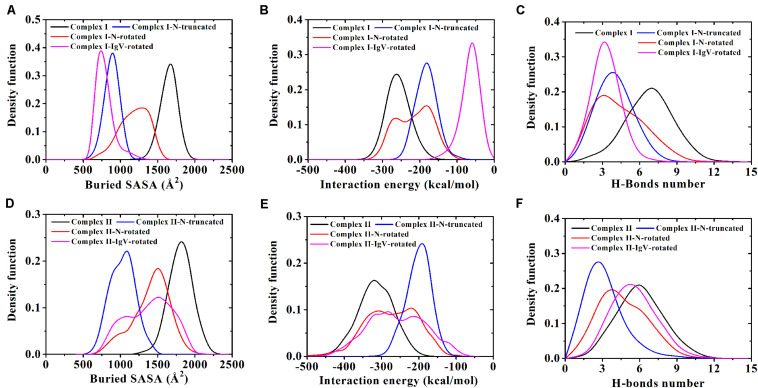
**(A–C)** Demonstrate distributions of buried SASA, interaction energy, and number of H-bonds for Complex I, Complex I-N-truncated, Complex I-N-rotated, and Complex I-IgV-rotated. **(D–F)** Demonstrate distributions of buried SASA, interaction energy, and number of H-bonds for Complex II, Complex II-N-truncated, Complex II-N-rotated, and Complex II-IgV-rotated.

Variations of buried SASA, interaction energy, and number of H-bonds were recorded for each complex to evaluate the stability of the complex interface (Figures 2, 3), and their distributions are shown in Figure 4. The buried SASA of Complex I fluctuated around 1,600 Å^2^, and its interaction energy and number of H-bonds fluctuated around −260 kcal/mol and 6, respectively (Figures 2A–C, 4A–C). However, the interface of Complex II was larger and stronger, with buried SASA, interaction energy and number of H-bonds fluctuating around 1,800 Å^2^, −320 kcal/mol and 6, respectively (Figures 3A–C, 4D–F).

H-bonds with a maximum survival ratio of above 0.2 were sorted out to recognize the interaction residues because they are proposed as the dominant linkers across the interface (Lee et al., 2016; Tan et al., 2017; Table 1). In total 15 H-bonds were detected for Complex I, and 20 for Complex II. Interaction residues on PD-1 of these two complexes all located on three loops, including the N-terminal loop (_PD–__1_ASP26, _PD–__1_PRO28, _PD–__1_ASP29, and _PD–__1_ARG30), the BC loop (_PD–__1_THR59, _PD–__1_SER60, and _PD–__1_GLU61), and the FG loop (_PD–__1_ALA129, _PD–__1_PRO130, and _PD–__1_LYS131) (Figures 2D–F, 3D–F). The FG loop formed five H-bonds in both the interfaces of Complex I and Complex II with the highest survival ratio of 0.68. The BC loop formed four and six H-bonds at the interface of Complex I and Complex II, respectively, with the highest survival ratio of 0.53. Although the numbers of H-bonds were similar to those of the FG loop, the interface formed by the BC loop was more vulnerable because it only appeared in two of three runs for both Complex I and Complex II. The average survival ratios of the H-bonds involved with the FG loop were higher than those with the BC loop, while the standard deviations with the FG loop were lower than those with the BC loop (Table 1). As the FG loop is a binding region for PD-L1, these results clearly suggest a stable steric clash blockade mechanism of nivolumab.

**TABLE 1:** Summary of survival ratio and involved residues of H-bonds in the interface of complex I and complex II.

**Complex**	**Bond No.**	**PD-1**			**Nivolumab**		**Survival ratio^#^**					
		**Secondary structure**	**Residue**	**Atom**	Residue*	**Atom**	**Equ1**	**Equ2**	**Equ3**	**Max**	**Ave**	**Std**
Complex I	1	N-terminal loop	ASP29	OD1	GLY33_H_	N	0.41	0.72	0.80	0.80	0.76	0.06
	2		ARG30	N	ASN31_H_	O	0.52	0.70	0.76	0.76	0.66	0.12
	3		ASP29	OD2	ASN99_H_	ND2	0.32	0.61	0.66	0.66	0.53	0.18
	4		ASP29	OD1	ASN99_H_	ND2	0.37	0.15	0.08	0.37	0.20	0.15
	5		ASP29	OD2	GLY33_H_	N	0.36	0.02	0.00	0.36	0.13	0.20
	6		PRO28	O	TYR53_H_	N	0.28	0.16	0.21	0.28	0.22	0.06
	7	FG loop	ALA129	O	THR56_L_	OG1	0.30	0.57	0.64	0.64	0.50	0.18
	8		PRO130	O	THR56_L_	N	0.50	0.62	0.56	0.62	0.56	0.06
	9		LYS131	NZ	ASP101_H_	OD2	0.36	0.27	0.29	0.36	0.31	0.05
	10		LYS131	N	ASP100_H_	O	0.35	0.28	0.32	0.35	0.32	0.04
	11		LYS131	NZ	ASP101_H_	OD1	0.26	0.32	0.31	0.32	0.30	0.03
	12	BC loop	GLU61	OE2	THR28_H_	OG1	0.01	0.43	0.39	0.43	0.28	0.23
	13		GLU61	OE2	THR28_H_	N	0.00	0.41	0.31	0.41	0.24	0.21
	14		GLU61	OE1	THR28_H_	OG1	0.01	0.32	0.31	0.32	0.21	0.18
	15		GLU61	OE1	THR28_H_	N	0.00	0.24	0.28	0.28	0.17	0.15
Complex II	16	N-terminal loop	ARG30	N	ASN31_H_	O	0.82	0.76	0.25	0.82	0.61	0.31
	17		ASP29	OD2	GLY33_H_	N	0.59	0.00	0.11	0.59	0.23	0.31
	18		ASP29	OD1	ASN99_H_	ND2	0.52	0.23	0.33	0.52	0.36	0.15
	19		ARG30	NH1	ASN31_H_	OD1	0.45	0.03	0.00	0.45	0.16	0.25
	20		ASP29	OD2	ASN99_H_	ND2	0.29	0.14	0.42	0.42	0.28	0.14
	21		ASP29	OD1	GLY33_H_	N	0.36	0.01	0.28	0.36	0.22	0.18
	22		PRO28	O	TYR53_H_	N	0.21	0.09	0.27	0.27	0.19	0.09
	23		ASP26	OD2	LYS57_H_	NZ	0.09	0.10	0.24	0.24	0.14	0.08
	24		ASP26	OD1	LYS57_H_	NZ	0.06	0.24	0.17	0.24	0.16	0.09
	25	FG loop	PRO130	O	THR56_L_	N	0.28	0.68	0.60	0.68	0.52	0.21
	26		LYS131	NZ	ASP101_H_	OD2	0.50	0.18	0.28	0.50	0.32	0.16
	27		LYS131	NZ	ASP101_H_	OD1	0.35	0.38	0.42	0.42	0.38	0.04
	28		ALA129	O	THR56_L_	OG1	0.08	0.29	0.06	0.29	0.14	0.13
	29		LYS131	NZ	ASN99_H_	O	0.25	0.15	0.16	0.25	0.19	0.06
	30	BC loop	THR59	O	THR28_H_	N	0.00	0.53	0.05	0.53	0.19	0.29
	31		GLU61	N	GLY26_H_	O	0.00	0.42	0.04	0.42	0.15	0.23
	32		GLU61	N	THR28_H_	OG1	0.01	0.00	0.32	0.32	0.11	0.18
	33		THR59	O	ASN31_H_	ND2	0.05	0.01	0.27	0.27	0.11	0.14
	34		SER60	OG	GLY26_H_	O	0.00	0.27	0.01	0.27	0.09	0.15
	35		THR59	OG1	ASN31_H_	ND2	0.01	0.21	0.03	0.21	0.08	0.11

*The name of the residues with H or L indicating that the residues are on the heavy or the light chain of nivolumab. ^#^Equ1, Equ2, and Equ3 donate three runs. Max represents the maximum value of three survival ratios of a bond. Ave represents the average value of three survival ratios of a bond. Std represents the standard deviation of three survival ratios of a bond.

In the crystal structures, the N-terminal loop of PD-1 is not a binding site for PD-L1 but forms a major interface with nivolumab. In our simulations, it involved in six H-bonds at the interface of Complex I and contributed nine H-bonds to the interface of Complex II, with the highest survival ratio of 0.82. The standard deviations of the survival ratios of the H-bonds formed by the N-terminal loop were a little high. This might be due to the high flexibility of the long N-terminal loop, which consists of eight residues (_PD–__1_SER27 to _PD–__1_PRO34) in Complex I and 10 residues (_PD–__1_LEU25 to _PD–__1_PRO34) in Complex II, and can only be constrained by one side. The_PD–__1_ASP26 located at the N-terminal loop formed two H-bonds with nivolumab in Complex II, but it was missing in Complex I. Thus, the N-terminal loop in Complex II formed a larger interface with nivolumab. These MD results showed that interactions between the N-terminus of PD-1 and nivolumab are definite and stable on the nanosecond time scale we simulated.

### Truncation of the N-Terminal Loop of PD-1 Impairs the Interface Between PD-1 and Nivolumab

The N-terminal loop of PD-1 greatly contributes to the interface of the nivolumab/PD-1 complex, and mutagenesis study revealed that the cut-off of the N-terminal loop abolished the binding between PD-1 and nivolumab. How will the truncation of the N-terminal loop impair the interfaces? To answer this question, we cut off the N-terminal loops in Complex I and II, and designated them as Complex I-N-truncated and Complex II-N-truncated (Figures 1B,F). These two systems were simulated with the same scenario as before. The RMSD of heavy atoms showed that these two complexes reached a local minimum after 20 ns (Supplementary Figures S3, S4).

**FIGURE 5:**
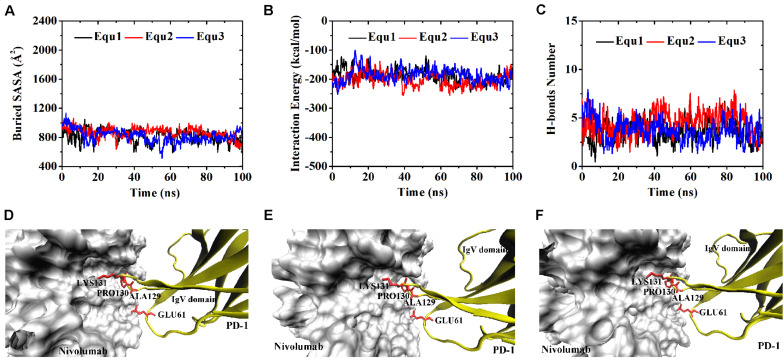
Buried SASA **(A)**, interaction energy **(B)**, and number of H-bonds **(C)** of Complex I-N-truncated in three runs (Equ1, Equ2, and Equ3). **(D–F)** Show the last frame of Complex I-N-truncated in Equ1, Equ2, and Equ3, respectively. The IgV domain of PD-1 is shown in yellow (New Cartoon mode). The interaction residues of PD-1 are shown in red (Licorice mode).

**FIGURE 6:**
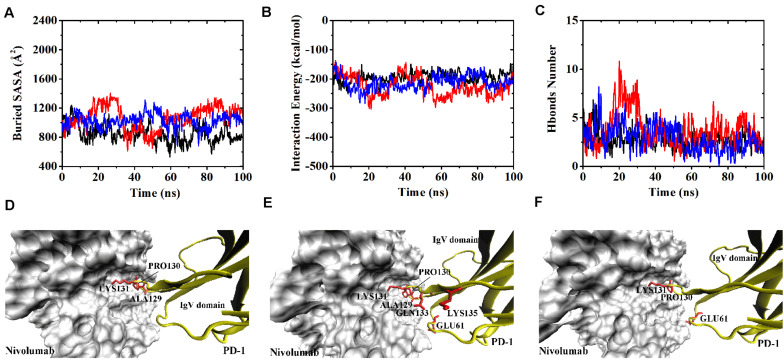
Buried SASA **(A)**, interaction energy **(B)**, and number of H-bonds **(C)** of Complex II-N-truncated in three runs (Equ1, Equ2, and Equ3). **(D–F)** Show the last frame of Complex II-N-truncated in Equ1, Equ2, and Equ3, respectively. The IgV domain of PD-1 is shown in yellow (New Cartoon mode). The interaction residues of PD-1 are shown in red (Licorice mode).

Variations of buried SASA, interaction energy, and number of H-bonds along the simulation time were analyzed, as shown in Figures 5A–C, 6A–C, and their distributions are shown in Figure 4. The buried SASA of Complex I-N-truncated greatly decreased to around 900 Å^2^, and its interaction energy and number of H-bonds dropped to -180 kcal/mol and 3, respectively. The buried SASA of Complex II-N-truncated greatly decreased to around 1,000 Å^2^, and its interaction energy and number of H-bonds dropped to -180 kcal/mol and 3, respectively. These results indicate that the truncation of the N-terminal loop seriously impairs the binding between PD-1 and nivolumab (Figures 5D–F, 6D–F). Moreover, the binding strength of Complex I-N-truncated is similar to that of Complex II-N-truncated, implying that Complex II is more stable than Complex I because it has a longer N-terminal loop (_PD–__1_LEU25 to _PD–__1_PRO34) than Complex I (_PD–__1_SER27 to _PD–__1_PRO34). This conclusion was further proved by the H-bonds with a survival ratio of above 0.2, where nine H-bonds for Complex I-N-truncated, and ten for Complex II-N-truncated were found with the interaction residues contributed by the FG and BC loops of PD-1, similar to those in Complex I and Complex II (Table 2).

**TABLE 2:** Summary of survival ratio and involved residues of H-bonds in the interface of complex I-N-truncated and complex II-N-truncated.

**Complex**	**Bond No.**	**PD-1**			**Nivolumab**		**Survival ratio^#^**					
		**Secondary structure**	**Residue**	**Atom**	Residue*	**Atom**	**Equ1**	**Equ2**	**Equ3**	**Max**	**Ave**	**Std**
Complex I-N-truncated	1	FG loop	PRO130	O	THR56_L_	N	0.77	0.74	0.71	0.77	0.74	0.03
	2		ALA129	O	THR56_L_	OG1	0.60	0.57	0.61	0.61	0.59	0.02
	3		LYS131	NZ	ASP101_H_	OD1	0.34	0.42	0.39	0.42	0.38	0.04
	4		LYS131	NZ	ASP101_H_	OD2	0.40	0.29	0.31	0.40	0.33	0.06
	5	BC loop	GLU61	OE1	TYR102_H_	OH	0.21	0.38	0.42	0.42	0.34	0.11
	6		GLU61	OE2	TYR102_H_	OH	0.30	0.25	0.38	0.38	0.31	0.07
	7		GLU61	OE1	THR28_H_	OG1	0.15	0.29	0.01	0.29	0.15	0.14
	8		GLU61	OE2	THR28_H_	OG1	0.08	0.26	0.14	0.26	0.16	0.09
	9		GLU61	N	GLY26_H_	O	0.18	0.23	0.20	0.23	0.20	0.03
Complex II-N-truncated	10	FG loop	PRO130	O	THR56_L_	N	0.75	0.47	0.71	0.75	0.64	0.15
	11		LYS131	NZ	ASP101_H_	OD1	0.52	0.46	0.33	0.52	0.44	0.10
	12		ALA129	O	THR56_L_	OG1	0.52	0.32	0.10	0.52	0.31	0.21
	13		LYS131	NZ	ASP101_H_	OD2	0.29	0.27	0.33	0.33	0.30	0.03
	14		LYS131	N	ASP100_H_	O	0.03	0.34	0.06	0.34	0.14	0.17
	15		LYS135	NZ	ASP100_H_	OD1	0.00	0.32	0.00	0.32	0.11	0.18
	16		LYS131	NZ	ASN99_H_	O	0.09	0.26	0.02	0.26	0.12	0.12
	17		GLN133	OE1	TYR49_L_	OH	0.03	0.20	0.05	0.20	0.09	0.09
	18	BC loop	GLU61	N	GLY26_H_	O	0.03	0.28	0.05	0.28	0.12	0.14
	19		GLU61	N	THR28_H_	OG1	0.00	0.02	0.22	0.22	0.08	0.12

*The name of the residues with H or L indicating that the residues are on the heavy or the light chain of nivolumab. ^#^Equ1, Equ2, and Equ3 donate three runs. Max represents the maximum value of three survival ratios of a bond. Ave represents the average value of three survival ratios of a bond. Std represents the standard deviation of three survival ratios of a bond.

**FIGURE 7:**
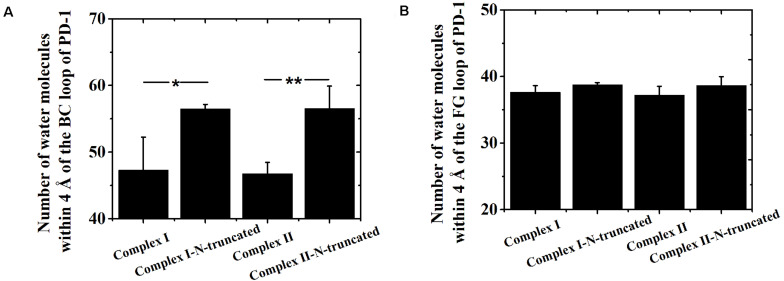
Number of water molecules within 4 Å of the BC **(A)** and FG **(B)** loops of Complex I, Complex I-N-truncated, Complex II and Complex II-N-truncated in three runs. *p < 0.05, **p < 0.01.

Although the interface of the nivolumab/PD-1 was seriously impaired by cutting off the N-terminal loop of PD-1, the dissociation was not observed. A possible reason is that the energy barrier involved in the FG and BC loops of PD-1 was too high to overcome within our simulation time. However, analysis of accessibility of water molecules around the FG and BC loops revealed one major difference before and after deletion of the N-terminal loop. As shown in Figures 7A,B, the difference lies in extra water accessibility near the BC loop, where about nine and ten more water molecules entered within 4 Å of the BC loops of Complex I-N-truncated and Complex II-N-truncated. For the FG loop, the water accessibility showed no significant change. This result suggests the interface involved in the BC loop is protected from water attack by the N-terminal loop. Deleting the N-terminal loop might lead to a fast dissociation of the BC loop from nivolumab.

### The N-Terminal Loop of PD-1 Prefers to Interact With Nivolumab to Stabilize the Complex Interface Further

Two binding regions were mapped on PD-1 for nivolumab, namely, the N-terminal loop and the IgV domain (including the FG loop and the BC loop), implying the possibility of a two-step binding process. Therefore, four additional complexes were built to verify this hypothesis. First, the N-terminal loop of PD-1 in Complex I and Complex II was rotated backward against the interface to dissociate from nivolumab to mimic the scenario where the IgV domain of PD-1 binds to nivolumab at the first step, designated as Complex I-N-rotated and Complex II-N-rotated, respectively (Figures 1C,G). Second, the IgV domain of PD-1 in Complex I and Complex II was rotated backward against the interface to dissociate from nivolumab to mimic the scenario where the N-terminal loop of PD-1 binds to nivolumab at the first step, designated as Complex I-IgV-rotated and Complex II-IgV-rotated, respectively (Figures 1D,H).

**FIGURE 8:**
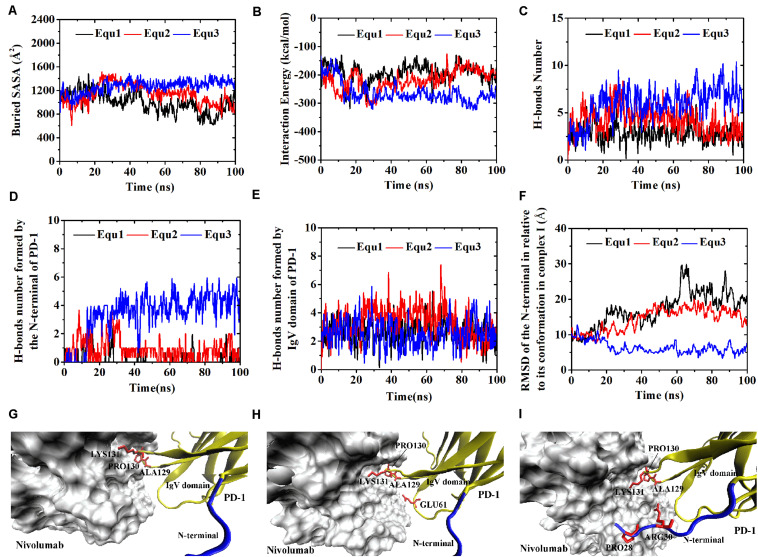
Buried SASA **(A)**, interaction energy **(B)**, and number of H-bonds **(C)** of Complex I-N-rotated in three runs (Equ1, Equ2, and Equ3). **(D,E)** Show the number of H-bonds formed by the N-terminal loop and the IgV domain of PD-1, respectively. **(F)** shows the RMSD of the N-terminal loop of PD-1 of Complex I-N-rotated in relative to its initial conformation in Complex I in three runs. **(G–I)** Show the last frame of Complex I-N-rotated in Equ1, Equ2, and Equ3, respectively. The N-terminal loop of PD-1 is shown in blue and the IgV domain of PD-1 is shown in yellow (New Cartoon mode). The interaction residues of PD-1 are shown in red (Licorice mode).

**FIGURE 9:**
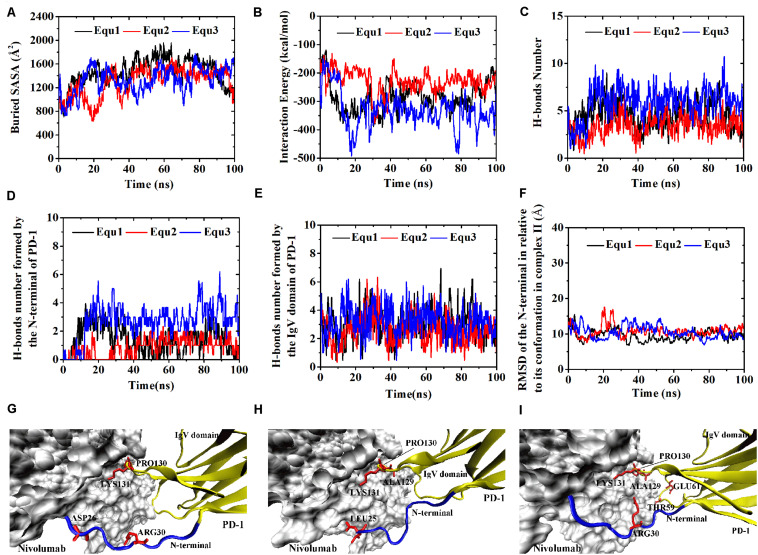
Buried SASA **(A)**, interaction energy **(B)**, and number of H-bonds **(C)** of Complex II-N-rotated in three runs (Equ1, Equ2, and Equ3). **(D,E)** Show the number of H-bonds formed by the N-terminal loop and the IgV domain of PD-1, respectively. **(F)** Shows the RMSD of the N-terminal loop of PD-1 of Complex II-N-rotated in relative to its initial conformation in Complex II in three runs. **(G–I)** Show the last frame of Complex II-N-rotated in Equ1, Equ2, and Equ3, respectively. The N-terminal loop of PD-1 is shown in blue and the IgV domain of PD-1 is shown in yellow (New Cartoon mode). The interaction residues of PD-1 are shown in red (Licorice mode).

Similarly, Complex I-N-rotated and Complex II-N-rotated were simulated for 100 ns thrice after an energy minimization of 10,000 steps. The RMSD of heavy atoms showed that these two complexes reached a local minimum after 20 ns (Supplementary Figures S5, S6). Buried SASA, interaction energy, and number of H-bonds of Complex I-N-rotated and Complex II-N-rotated are shown in Figures 8A–C, 9A–C, respectively, and their distributions are demonstrated in Figure 4. For Complex I-N-rotated, its buried SASA decreased to around 800 Å^2^ during Equ1 and Equ2, and its interaction energy and number of H-bonds decreased to around -180 kcal/mol and 3, respectively, which was close to the binding strength of Complex I-N-truncated. However, its buried SASA increased to around 1,400 Å^2^ during Equ3 with interaction energy and number of H-bonds fluctuating around −280 kcal/mol and 7, respectively, which was close to the binding strength of the initial Complex I.

Were these changes induced by the N-terminal loop or the IgV domain of PD-1? To answer this question, the number of intermolecular H-bonds formed by the N-terminal loop and the IgV domain of PD-1 was calculated (Figures 8D,E). H-bonds with a survival ratio of above 0.2 are listed in Table 3. The results clearly demonstrated that the increase of the complex interface was mainly caused by the N-terminal loop. The RMSD of the N-terminal loop in relative to its initial conformation in Complex I further confirmed that it returned back toward nivolumab in Equ3 (Figure 8F). The N-terminal loop bound to nivolumab with six H-bonds (Bond No. 1–6 in Table 3) formed by _PD–__1_ARG30 and _PD–__1_PRO28 after 10 ns in Equ3, but it kept free in Equ1 and Equ2 until the end of simulations (Figures 8G–I).

**TABLE 3:** Summary of survival ratio and involved residues of H-bonds in the interface of complex I-N-rotated and complex II-N-rotated

**Complex**	**Bond No.**	**PD-1**			**Nivolumab**		**Survival ratio^#^**					
		**Secondary structure**	**Residue**	**Atom**	Residue*	**Atom**	**Equ1**	**Equ2**	**Equ3**	**Max**	**Ave**	**Std**
Complex I-N-rotated	1	N-terminal loop	ARG30	NH1	ASN31_H_	O	0.00	0.00	0.75	0.75	0.25	0.43
	2		PRO28	O	TYR53_H_	OH	0.00	0.01	0.60	0.60	0.20	0.34
	3		ARG30	NH1	ASP100_H_	OD1	0.02	0.03	0.54	0.54	0.20	0.30
	4		ARG30	NH2	ASP100_H_	OD2	0.02	0.09	0.48	0.48	0.20	0.25
	5		ARG30	NH2	ASP100_H_	OD1	0.00	0.05	0.24	0.24	0.10	0.13
	6		ARG30	NH1	ASP100_H_	OD2	0.01	0.02	0.23	0.23	0.09	0.12
	7	FG loop	PRO130	O	THR56_L_	N	0.65	0.71	0.65	0.71	0.67	0.03
	8		ALA129	O	THR56_L_	OG1	0.41	0.53	0.43	0.53	0.46	0.06
	9		LYS131	NZ	ASP101_H_	OD1	0.27	0.44	0.49	0.49	0.40	0.12
	10		LYS131	NZ	ASP101_H_	OD2	0.48	0.21	0.25	0.48	0.31	0.15
	11		LYS131	NZ	ASN99_H_	O	0.14	0.13	0.23	0.23	0.17	0.06
	12	BC loop	GLU61	N	GLY26_H_	O	0.00	0.43	0.00	0.43	0.14	0.25
	13		GLU61	OE1	TYR102_H_	OH	0.01	0.39	0.00	0.39	0.13	0.22
	14		GLU61	OE2	TYR102_H_	OH	0.03	0.25	0.00	0.25	0.09	0.14
Complex II-N-rotated	15	N-terminal loop	ARG30	NH1	ASN31_H_	O	0.00	0.00	0.62	0.62	0.21	0.36
	16		ARG30	NE	ASN31_H_	OD1	0.52	0.00	0.00	0.52	0.17	0.30
	17		LEU25	O	TYR53_H_	OH	0.00	0.52	0.00	0.52	0.17	0.30
	18		ARG30	NH1	ASP100_H_	OD2	0.00	0.00	0.41	0.41	0.14	0.24
	19		ARG30	NH2	ASP100_H_	OD1	0.00	0.00	0.40	0.40	0.13	0.23
	20		ARG30	NH1	ASP100_H_	OD1	0.00	0.01	0.38	0.38	0.13	0.22
	21		ARG30	NH2	ASP100_H_	OD2	0.00	0.00	0.38	0.38	0.13	0.22
	22		ASP26	OD1	LYS57_H_	NZ	0.24	0.02	0.05	0.24	0.10	0.12
	23	FG loop	PRO130	O	THR56_L_	N	0.62	0.78	0.81	0.81	0.74	0.10
	24		LYS131	NZ	ASP101_H_	OD2	0.43	0.32	0.50	0.50	0.42	0.09
	25		ALA129	O	THR56_L_	OG1	0.14	0.32	0.40	0.40	0.29	0.13
	26		LYS131	NZ	ASP101_H_	OD1	0.19	0.20	0.32	0.32	0.24	0.07
	27		LYS131	N	ASP100_H_	O	0.26	0.11	0.03	0.26	0.13	0.12
	28	BC loop	GLU61	N	GLN1_H_	OY	0.00	0.00	0.27	0.27	0.09	0.16
	29		THR59	OG1	GLY26_H_	O	0.00	0.00	0.22	0.22	0.07	0.13

*The name of the residues with H or L indicating that the residues are on the heavy or the light chain of nivolumab. ^#^Equ1, Equ2, and Equ3 donate three runs. Max represents the maximum value of three survival ratios of a bond. Ave represents the average value of three survival ratios of a bond. Std represents the standard deviation of three survival ratios of a bond.

The buried SASA of Complex II-N-rotated increased to around 1500 Å^2^ in all three runs (Figures 9A–C). Its interaction energy decreased to around −350 kcal/mol in Equ1 and Equ3, and to around −250 kcal/mol in Equ2. The number of H-bonds of Complex II-N-rotated increased to 4 in Equ1 and Equ2, but to 6 in Equ3. Next, the number of H-bonds formed by the N-terminal loop as well as its RMSD in relative to its initial conformation in Complex II were calculated, as shown in Figures 9D–F and H-bonds with a survival ratio of above 0.2 are listed in Table 3. The results reveal that the N-terminal loop rebuilt the complex interface and formed stable H-bonds with nivolumab in all three runs after 20 ns, especially in Equ3. _PD–__1_ARG30 and _PD–__1_ASP26 on the N-terminal loop formed two H-bonds with ASN31_H_ and LYS57_H_ of nivolumab (Bond No. 16 and 22 in Table 3) in Equ1, whereas _PD–__1_LEU25 formed one bond with TYR53_H_ in Equ2 (Bond No. 17 in Table 3). The interface between the N-terminal loop of PD-1 and the nivolumab in Equ3 was most stable, with five H-bonds formed by _PD–__1_ARG30 with ASN31_H_ and ASP100_H_ of nivolumab (Bond No. 15, 18–21 in Table 3 and Figures 9G–I).

Overall, on the nanosecond time scale, the N-terminal loop of PD-1 prefers to interact with nivolumab to stabilize the complex interface further. Interfaces of Complex I-N-rotated and Complex II-N-rotated tend to be stronger with the help of the N-terminal loop of PD-1. The binding strength indexes showed bimodal distributions, especially for the interaction energy of Complex II-N-rotated (red lines in Figure 4).

### Binding of the N-Terminal Loop With Nivolumab Could Induce the Rebinding of the IgV Domain With Nivolumab

**FIGURE 10:**
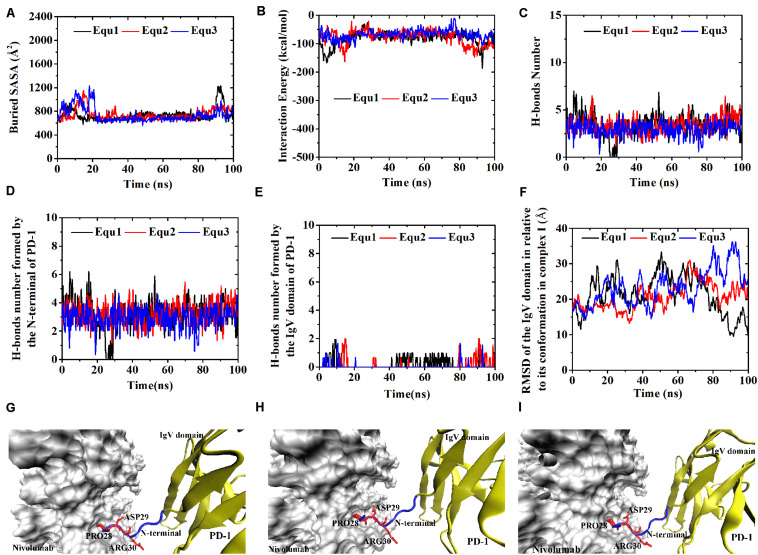
Buried SASA **(A)**, interaction energy **(B)**, and number of H-bonds **(C)** of Complex I-IgV-rotated in three runs (Equ1, Equ2, and Equ3). **(D,E)** Show the number of H-bonds formed by the N-terminal loop and the IgV domain of PD-1, respectively. **(F)** Shows the RMSD of the IgV domain of PD-1 of Complex I-IgV-rotated in relative to its initial conformation in Complex I in three runs. **(G–I)** Show the last frame of Complex I-IgV-rotated in Equ1, Equ2, and Equ3, respectively. The N-terminal loop of PD-1 is shown in blue and the IgV domain of PD-1 is shown in yellow (New Cartoon mode). The interaction residues of PD-1 are shown in red (Licorice mode).

**FIGURE 11:**
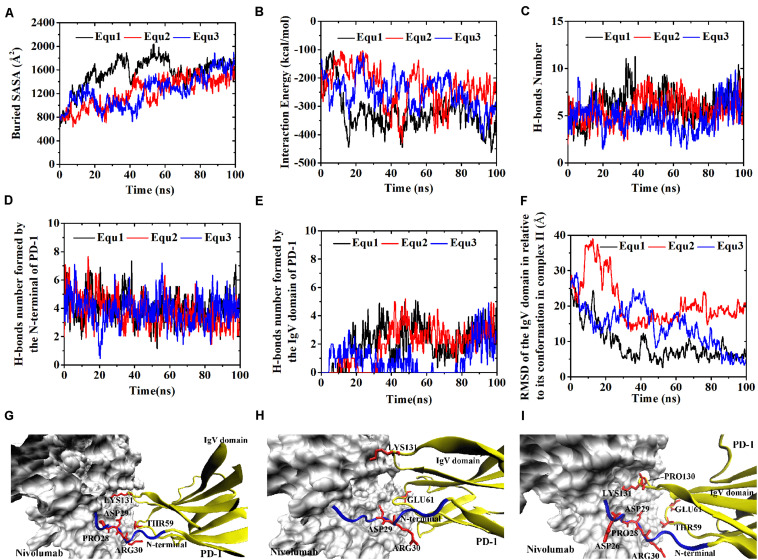
Buried SASA **(A)**, interaction energy **(B)**, and number of H-bonds **(C)** of Complex II-IgV-rotated in three runs (Equ1, Equ2, and Equ3). **(D,E)** Show the number of H-bonds formed by the N-terminal loop and the IgV domain of PD-1, respectively. **(F)** Shows the RMSD of the IgV domain of PD-1 of Complex II-IgV-rotated in relative to its initial conformation in Complex II in three runs. **(G–I)** Show the last frame of Complex II-IgV-rotated in Equ1, Equ2, and Equ3, respectively. The N-terminal loop of PD-1 is shown in blue and the IgV domain of PD-1 is shown in yellow (New Cartoon mode). The interaction residues of PD-1 are shown in red (Licorice mode).

For the IgV-rotated complexes, the RMSD of heavy atoms showed that Complex I-IgV-rotated fluctuated more violently than Complex II-IgV-rotated in three runs (Supplementary Figures S7, S8). Buried SASA, interaction energy, and number of H-bonds of Complex I-IgV-rotated and Complex II-IgV-rotated are shown in Figures 10A–C, 11A–C, respectively, and their distributions are demonstrated in Figure 4. For all three runs of Complex I-IgV-rotated, the buried SASA fluctuated around 700 Å^2^, the interaction energy fluctuated around −70 kcal/mol, and the number of H-bonds fluctuated around 3 (Figures 10A–C). The interface of complex I-IgV-rotated was mainly contributed by the N-terminal loop of the PD-1 within our simulation time (Figures 10D,E), with seven H-bonds formed by _PD–__1_PRO28, _PD–__1_ASP29, and _PD–__1_ARG30 with ASN31_H_, GLY33_H_, TYR53_H_, and ASN99_H_ of nivolumab (Bond No. 1–7 in Table 4 and Figures 10G–I). The RMSD of the IgV domain in relative to its initial conformation in Complex I showed that the IgV domain of PD-1 remained free in all three runs, which caused great fluctuations (Figure 10F).

**TABLE 4:** Summary of survival ratio and involved residues of H-bonds in the interface of complex I-IgV-rotated and complex II-IgV-rotated.

**Complex**	**Bond No.**	**PD-1**			**Nivolumab**		**Survival ratio^#^**					
		**Secondary structure**	**Residue**	**Atom**	Residue*	**Atom**	**Equ1**	**Equ2**	**Equ3**	**Max**	**Ave**	**Std**
Complex I-IgV-rotated	1	N-terminal loop	ASP29	OD1	GLY33_H_	N	0.22	0.41	0.95	0.95	0.53	0.38
	2		ARG30	N	ASN31_H_	O	0.84	0.82	0.71	0.84	0.79	0.07
	3		ASP29	OD2	GLY33_H_	N	0.68	0.49	0.00	0.68	0.39	0.35
	4		ASP29	OD2	ASN99_H_	ND2	0.23	0.44	0.64	0.64	0.44	0.21
	5		ASP29	OD1	ASN99_H_	ND2	0.55	0.41	0.22	0.55	0.39	0.17
	6		PRO28	O	TYR53_H_	N	0.25	0.29	0.29	0.29	0.28	0.02
	7		ARG30	NH1	ASN31_H_	OD1	0.20	0.15	0.05	0.20	0.13	0.08
Complex II-IgV-rotated	8	N-terminal loop	ASP29	OD1	GLY33_H_	N	0.95	0.06	0.24	0.95	0.42	0.47
	9		ASP29	OD2	GLY33_H_	N	0.00	0.90	0.70	0.90	0.53	0.47
	10		ARG30	N	ASN31_H_	O	0.84	0.82	0.80	0.84	0.82	0.02
	11		ASP29	OD1	ASN99_H_	ND2	0.17	0.67	0.49	0.67	0.44	0.25
	12		ASP29	OD2	ASN99_H_	ND2	0.63	0.17	0.31	0.63	0.37	0.24
	13		ARG30	NH1	ASN31_H_	OD1	0.19	0.04	0.36	0.36	0.20	0.16
	14		PRO28	O	TYR53_H_	N	0.26	0.19	0.29	0.29	0.25	0.05
	15		ARG30	NE	ASN31_H_	OD1	0.28	0.14	0.06	0.28	0.16	0.11
	16		ASP26	OD1	LYS57_H_	NZ	0.07	0.09	0.20	0.20	0.12	0.07
	17	FG loop	LYS131	NZ	ASP101_H_	OD1	0.49	0.00	0.01	0.49	0.17	0.28
	18		LYS131	NZ	ASN99_H_	O	0.28	0.00	0.09	0.28	0.12	0.14
	19		LYS131	NZ	ASP101_H_	OD2	0.22	0.00	0.26	0.26	0.16	0.14
	20		PRO130	O	THR56_L_	N	0.18	0.00	0.21	0.21	0.13	0.11
	21		LYS131	NZ	ASP50_L_	OD1	0.00	0.20	0.01	0.20	0.07	0.11
	22	BC loop	GLU61	OE1	THR28_H_	OG1	0.08	0.32	0.00	0.32	0.13	0.17
	23		GLU61	OE2	THR28_H_	N	0.03	0.31	0.00	0.31	0.11	0.17
	24		GLU61	OE1	THR28_H_	N	0.02	0.25	0.00	0.25	0.09	0.14
	25		GLU61	OE2	THR28_H_	OG1	0.13	0.23	0.01	0.23	0.12	0.11
	26		THR59	O	ASN31_H_	ND2	0.22	0.06	0.13	0.22	0.14	0.08

*The name of the residues with H or L indicating that the residues are on the heavy or the light chain of nivolumab. ^#^Equ1, Equ2, and Equ3 donate three runs. Max represents the maximum value of three survival ratios of a bond. Ave represents the average value of three survival ratios of a bond. Std represents the standard deviation of three survival ratios of a bond.

By contrast, for all three runs of Complex II-IgV-rotated, the buried SASA increased to nearly 1,600 Å^2^, the interaction energy decreased to nearly −300 kcal/mol, and the number of H-bonds increased to around 6. The number of H-bonds formed by the N-terminal loop and the IgV domain of PD-1, as well as the RMSD of the IgV domain in relative to its initial conformation in Complex II are shown in Figures 11D–F. It can be seen that the N-terminal loop of PD-1 maintained a stable interface with nivolumab on the nanosecond time scale, and changes were mainly caused by the IgV domain, which got close to and formed stable interface with nivolumab in all three runs. The FG loop (_PD–__1_LYS131) and BC loop (_PD–__1_THR59) of PD-1 interacted firmly with nivolumab (ASN31_H_, ASN99_H_, and ASP101_H_) by forming four bonds in Equ1 (Bond No. 17–19, 26 in Table 4 and Figure 11G). _PD–__1_LYS131 of FG loop and _PD–__1_GLU61 of BC loop formed five H-bonds with ASP50_L_ and THR28_H_ of nivolumab in Equ2 (Bond No. 21–25 in Table 4 and Figure 11H). The FG loop (i.e., _PD–__1_LYS131 and _PD–__1_PRO130) formed two H-bonds with ASP101_H_ and THR56_L_ of nivolumab in Equ3 (Bond No. 19–20, Figure 11I). Thus, in our simulations, binding of the N-terminal loop with nivolumab could further induce the interaction of the IgV domain with nivolumab, which would switch the interface of nivolumab/PD-1 complex to a stronger binding state (purple lines in Figures 4D–F).

## Discussion

The N-terminal loop of PD-1 has not attracted much attention in recent years because it is not a binding region for PD-L1. However, it was proven critical for the binding between PD-1 and nivolumab, which is a humanized IgG4 antibody approved by the FDA for several cancers. Crystal structures of nivolumab/PD-1 complex showed that the interaction residues of PD-1 locate on the N-terminal loop and IgV domain. The N-terminal loop of PD-1 greatly contributes to the complex formation, and we believe that it should also play an important role in the dynamic molecular recognition process. As dynamics of terminal loops are hard to predict based on crystal structure alone, eight molecular systems of nivolumab/PD-1 complex with different binding states were established, and long-time MD simulations of three replicas were performed for each of them, with the total simulated time of 2.4 μs. Our results proposed a two-step binding mode, in which the N-terminal loop of PD-1 could switch the complex interface into a stronger binding state. When the IgV domain binds to nivolumab first, the N-terminal loop of PD-1 prefers to interact with nivolumab to stabilize the complex interface. When the N-terminal loop is occupied by nivolumab, it could further induce the binding between the IgV domain (i.e., the FG and BC loops) of PD-1 and nivolumab.

The present results provided a detailed picture on the dynamic properties of the N-terminal loop of PD-1 in molecular interactions. Although this is the first time to systematically study the function of the N-terminal loop of PD-1, the N-terminal loops of other proteins have been revealed to have similar regulatory functions. For example, the N-terminus of model protein thaumatin serves as a major binding for cisplatin fragments (Russo Krauss et al., 2016). The N-terminal loop residues of beta-amyloid plays a key role in its interactions with integrin receptor and cell surface (Venkatasubramaniam et al., 2014). The N-terminal loop region of A1 domain in von Willebrand factor could stabilize A1A2A3 complex and modulate platelet activation under shear stress (Ju et al., 2013). Surface-exposed loops, generally as the most flexible parts of a protein structure, are not mere connectors but also have the potential to interact with solvent, ligands, and other biomolecules (Papaleo et al., 2016).

Since loop regions are too flexible to be resolved by crystallography, our simulations pave the way for investigating the binding mechanism between PD-1 and nivolumab. With the proposed two-step binding mode, nivolumab might be at least twice as likely to bind PD-1 as other antibodies with only one binding site. Furthermore, due to the high flexibility and mobility of the N-terminal loop, it can greatly facilitate the scanning efficiency and thus increase the probability of PD-1-nivolumab binding. This is of great importance for molecular interactions in the crowded intracellular environment.

Besides the binding process, our work also revealed the role of the N-terminal loop in augmenting the PD-1-nivolumab residence time, which is defined as the reciprocal of the dissociation rate constant. An abundance of experimental data suggests that high-affinity drug interactions with macromolecular targets generally rely on multistep binding and dissociation described by the two-step, induced-fit model (Copeland, 2016). Here, we show that the dissociation trajectory for the PD-1/nivolumab complex probably involves a retrograde induced-fit mechanism, that the N-terminal loop of PD-1 is able to rebind the dissociated nivolumab and IgV domain before nivolumab is completely released from PD-1 (Figure 11). Thus, the retrograde induced-fit mechanism creates multiple kinetic and structural barriers to nivolumab dissociation. This might partially explain why nivolumab has a nearly 10-fold higher affinity than that of pembrolizumab (Kd = 3.06 vs. 29 pM) (Fessas et al., 2017), another humanized anti-PD-1 monoclonal antibody approved by FDA, although the epitope of nivolumab is distinctly smaller than that of pembrolizumab (buried surface areas = 1,487 vs. 2,126 Å^2^). This model suggests that the N-terminal loop of PD-1 may be viewed as a “kinetic gatekeeper” that guides the docking of nivolumab onto the IgV domain and prevents nivolumab from dissociating.

Apart from the role of pulling the IgV domain back to nivolumab, the N-terminal loop may also increase the nivolumab residence time by shielding the IgV-nivolumab H-bonds from water. In previously reported studies, the relationship between long residence time and accessibility of water has been established (Schmidtke et al., 2011; Magarkar et al., 2019). In this study, binding of the N-terminal loop with nivolumab can create an environment of lower dielectric constant around the BC loop, shielded from bulk solvent (Figure 7). Such a solvent-shielded environment might result in the strengthening of non-covalent forces between the BC loop and nivolumab, such as hydrogen bonds, hydrophobic forces and van der Waals forces.

The function of the N-terminal loop of PD-1 for nivolumab requires its structure to be as complete as possible. The N-terminal loop of PD-1 in Complex I-N-rotated only bound with nivolumab in one run (Equ3 for complex I-N-rotated, Figure 8I), and it did not induce the rebinding of the IgV domain with nivolumab in the simulations (Figure 10). By contrast, the N-terminal loop of PD-1 interacted with nivolumab in all three runs of Complex II-N-rotated (Figure 9) and successfully induced the binding of the IgV domain with nivolumab in the simulations of Complex II-IgV-rotated (Figure 11). The reason is that the N-terminal loop of PD-1 contained 10 residues (_PD–__1_LEU25 to _PD–__1_PRO34) in Complex II, but it only eight residues (_PD–__1_SER27 to _PD–__1_PRO34) in Complex I. The missing residues in Complex I, such as _PD–__1_LEU25 and _PD–__1_ASP26, could also form stable H-bonds with nivolumab (Bond No. 23–24 in Table 1, Bond No. 17 and 22 in Table 3, and Bond No. 16 in Table 4). Therefore, the N-terminal loop of PD-1 in Complex II-N-rotated is more likely to be captured by nivolumab. Moreover, the interface between the N-terminal loop of PD-1 and nivolumab in Complex II-IgV-rotated (around 800 Å^2^ and -140 kcal/mol) was stronger than that in Complex I-IgV-rotated (around 700 Å^2^ and −70 kcal/mol), which is more beneficial in pulling the IgV domain back to nivolumab.

Despite the use of massive computational resources and highly precise models (full atomic representation and detailed force field), plain all-atom MD simulation is still insufficient for adequately exploring biomolecular structural dynamics. Multiple evidences indicate that long simulations cannot address how to catch the possible transition paths, which are still rare during μs-long MD due to inherent stochasticity and high-energy barriers. Nevertheless, we may predict some approximate behavior from simulations that suffer from sampling inefficiencies, in certain conditions e.g., upon introducing mutations or relaxation after removing ligands (Orellana, 2019). Here, we employed different starting configurations and multiple short simulations to enhances the configuration space sampling to better probe the conformational changes (Knapp et al., 2018; Chen et al., 2019b,c; Chen et al., 2020). Fortunately, the conformational transitions of the nivolumab/PD-1 complex we concerned have been observed in a simulation time of 100 ns. For example, the N-terminal loop of PD-1 rotated back toward the interface and interacted with nivolumab in all three runs of Complex I-N-rotated (Figure 9). Moreover, the IgV domain of PD-1 also rotated back toward the interface and interacted with nivolumab with the help of the N-terminal loop of PD-1 in the simulations of Complex II-IgV-rotated (Figure 11). This work provides useful dynamics information on the role of the N-terminal loop in the molecular recognition process between PD-1 and nivolumab.

Previous research usually focused on the “hot-spot” of PD-1, such as the FG loop and the C’D loop. Our results suggest that the N-terminal loop of PD-1, which acts as an important gatekeeper for the binding of nivolumab and PD-1, should also be considered in the anti-PD-1 blockade antibody design. We are hopeful that the results presented in this study will ultimately provide a theoretical framework to understand the structural landscape of N-terminal loop of PD-1. In general, this opens a new opportunity for medicinal biologists or chemists to optimize affinity for antibodies, if such gatekeepers can be identified.

## Data Availability Statement

All datasets presented in this study are included in the article/Supplementary Material.

## Author Contributions

GL and WL conceived and designed the experiments and wrote the manuscript. WL, HJ, and TC conducted modeling and simulation. WL, SL, and GZ analyzed the results. All authors contributed to the article and approved the submitted version.

## Conflict of Interest

The authors declare that the research was conducted in the absence of any commercial or financial relationships that could be construed as a potential conflict of interest.

## References

[B1] Bas D. C. Rogers D. M. Jensen J. H. (2008). Very fast prediction and rationalization of pKa values for protein-ligand complexes. *Proteins Struct. Funct. Bioinformatics* 73 765–783. 10.1002/prot.22102 18498103

[B2] Bellmunt J. Powles T. Vogelzang N. J. (2017). A review on the evolution of PD-1/PD-L1 immunotherapy for bladder cancer: the future is now. *Cancer Treat. Rev.* 54 58–67. 10.1016/j.ctrv.2017.01.007 28214651

[B3] Best R. B. Zhu X. Shim J. Lopes P. E. Mittal J. Feig M. (2012). Optimization of the additive CHARMM all-atom protein force field targeting improved sampling of the backbone phi, psi and side-chain chi(1) and chi(2) dihedral angles. *J. Chem. Theory Comput.* 8 3257–3273. 10.1021/ct300400x 23341755 PMC3549273

[B4] Biasini M. Bienert S. Waterhouse A. Arnold K. Studer G. Schmidt T. (2014). SWISS-MODEL: modelling protein tertiary and quaternary structure using evolutionary information. *Nucleic Acids Res.* 42 W252–W258. 10.1093/nar/gku340 24782522 PMC4086089

[B5] Bordoli L. Kiefer F. Arnold K. Benkert P. Battey J. Schwede T. (2008). Protein structure homology modeling using SWISS-MODEL workspace. *Nat. Protoc.* 4 1–13. 10.1038/nprot.2008.197 19131951

[B6] Bray F. Ferlay J. Soerjomataram I. Siegel R. L. Torre L. A. Jemal A. (2018). Global cancer statistics 2018: GLOBOCAN estimates of incidence and mortality worldwide for 36 cancers in 185 countries. *CA Cancer J. Clin.* 68 394–424. 10.3322/caac.21492 30207593

[B7] Callahan M. K. Postow M. A. Wolchok J. D. (2016). Targeting T Cell Co-receptors for Cancer therapy. *Immunity* 44 1069–1078. 10.1016/j.immuni.2016.04.023 27192570

[B8] Chen D. Tan S. Zhang H. Wang H. He W. Shi R. (2019a). The FG Loop of PD-1 Serves as a “Hotspot” for therapeutic monoclonal antibodies in tumor immune checkpoint therapy. *iScience* 14 113–124. 10.1016/j.isci.2019.03.017 30952089 PMC6447726

[B9] Chen J. Wang X. Pang L. Zhang J. Z. H. Zhu T. (2019b). Effect of mutations on binding of ligands to guanine riboswitch probed by free energy perturbation and molecular dynamics simulations. *Nucleic Acids Res.* 47 6618–6631. 10.1093/nar/gkz499 31173143 PMC6649850

[B10] Chen J. Wang J. Yin B. Pang L. Wang W. Zhu W. (2019c). Molecular mechanism of binding selectivity of inhibitors toward BACE1 and BACE2 Revealed by multiple short molecular dynamics simulations and free-energy predictions. *ACS Chem. Neurosci.* 10 4303–4318. 10.1021/acschemneuro.9b00348 31545898

[B11] Chen J. Liu X. Zhang S. Sun H. Zhang L. Zhang Q. (2020). Molecular mechanism with regard to the binding selectivity of inhibitors toward FABP5 and FABP7 explored by multiple short molecular dynamics simulations and free energy analyses. *Phys. Chem. Chem. Phys.* 22 2262–2275. 10.1039/c9cp05704h 31917380

[B12] Copeland R. A. (2016). The drug-target residence time model: a 10-year retrospective. *Nat. Rev. Drug Discov.* 15 87–95. 10.1038/nrd.2015.18 26678621

[B13] Couzin-Frankel J. (2013). Breakthrough of the year 2013. Cancer Immunotherapy. *Science* 342 1432–1433. 10.1126/science.342.6165.1432 24357284

[B14] Fang X. Fang Y. Liu L. Liu G. Wu J. (2012). Mapping paratope on antithrombotic antibody 6B4 to epitope on platelet glycoprotein Ibalpha via molecular dynamic simulations. *PLoS One* 7:e42263. 10.1371/journal.pone.0042263 22860101 PMC3408434

[B15] Fessas P. Lee H. Ikemizu S. Janowitz T. (2017). A molecular and preclinical comparison of the PD-1-targeted T-cell checkpoint inhibitors nivolumab and pembrolizumab. *Semin. Oncol.* 44 136–140. 10.1053/j.seminoncol.2017.06.002 28923212 PMC5612055

[B16] Guo Z. S. (2018). The 2018 Nobel Prize in medicine goes to cancer immunotherapy (editorial for BMC cancer). *BMC Cancer* 18:1086. 10.1186/s12885-018-5020-3 30415640 PMC6231274

[B17] Hirano F. Kaneko K. Tamura H. Dong H. Wang S. Ichikawa M. (2005). Blockade of B7-H1 and PD-1 by monoclonal antibodies potentiates cancer therapeutic immunity. *Cancer Res.* 65 1089–1096. 15705911

[B18] Humphrey W. Dalke A. Schulten K. (1996). VMD: visual molecular dynamics. *J. Mol. Graph.* 14 33–38, 27–38. 10.1016/0263-7855(96)00018-5 8744570

[B19] Ivashko I. N. Kolesar J. M. (2016). Pembrolizumab and nivolumab: PD-1 inhibitors for advanced melanoma. *Am. J. Health Syst. Pharm.* 73 193–201. 10.2146/ajhp140768 26843495

[B20] Ju L. Dong J. F. Cruz M. A. Zhu C. (2013). The N-terminal flanking region of the A1 domain regulates the force-dependent binding of von Willebrand factor to platelet glycoprotein Ibalpha. *J. Biol. Chem.* 288 32289–32301. 10.1074/jbc.M113.504001 24062306 PMC3820866

[B21] Kar G. Kuzu G. Keskin O. Gursoy A. (2012). Protein-protein interfaces integrated into interaction networks: implications on drug design. *Curr. Pharm. Des.* 18 4697–4705. 10.2174/138161212802651643 22650259

[B22] Keir M. E. Butte M. J. Freeman G. J. Sharpe A. H. (2008). PD-1 and its ligands in tolerance and immunity. *Annu. Rev. Immunol.* 26 677–704. 10.1146/annurev.immunol.26.021607.090331 18173375 PMC10637733

[B23] Kim E. S. (2017). Avelumab: first global approval. *Drugs* 77 929–937. 10.1007/s40265-017-0749-6 28456944

[B24] Knapp B. Ospina L. Deane C. M. (2018). Avoiding false positive conclusions in molecular simulation: the importance of replicas. *J. Chem. Theory Comput.* 14 6127–6138. 10.1021/acs.jctc.8b00391 30354113

[B25] Lee J. Y. Lee H. T. Shin W. Chae J. Choi J. Kim S. H. (2016). Structural basis of checkpoint blockade by monoclonal antibodies in cancer immunotherapy. *Nat. Commun.* 7:13354. 10.1038/ncomms13354 27796306 PMC5095608

[B26] Leventakos K. Mansfield A. S. (2016). Advances in the treatment of non-small cell lung cancer: focus on nivolumab. Pembrolizumab, and Atezolizumab. *BioDrugs* 30 397–405. 10.1007/s40259-016-0187-0 27411930

[B27] Liu W. Huang B. Kuang Y. Liu G. (2017). Molecular dynamics simulations elucidate conformational selection and induced fit mechanisms in the binding of PD-1 and PD-L1. *Mol. Biosyst.* 13 892–900. 10.1039/c7mb00036g 28327740

[B28] Liu W. Liu G. Zhou H. Fang X. Fang Y. Wu J. (2016). Computer prediction of paratope on antithrombotic antibody 10B12 and epitope on platelet glycoprotein VI via molecular dynamics simulation. *Biomed. Eng. Online* 15(Suppl. 2):152. 10.1186/s12938-016-0272-0 28155721 PMC5260068

[B29] Liu X. Shi D. Zhou S. Liu H. Yao X. (2018). Molecular dynamics simulations and novel drug discovery. *Expert Opin. Drug Discov.* 13 23–37. 10.1080/17460441.2018.1403419 29139324

[B30] Liu W. Liu G. (2017). “Mapping paratope and epitope residues of antibody pembrolizumab via molecular dynamics simulation,” in *Bioinformatics Research and Applications. ISBRA 2017. Lecture Notes in Computer Science*, Vol. 10330, eds Z. Cai, O. Daescu, and M. Li (Berlin: Springer). 10.1007/978-3-319-59575-7_11

[B31] Magarkar A. Schnapp G. Apel A. K. Seeliger D. Tautermann C. S. (2019). Enhancing drug residence time by shielding of intra-protein hydrogen bonds: a case study on CCR2 antagonists. *ACS Med. Chem. Lett.* 10 324–328. 10.1021/acsmedchemlett.8b00590 30891134 PMC6421533

[B32] Mellman I. Coukos G. Dranoff G. (2011). Cancer immunotherapy comes of age. *Nature* 480 480–489. 10.1038/nature10673 22193102 PMC3967235

[B33] Miller K. D. Nogueira L. Mariotto A. B. Rowland J. H. Yabroff K. R. Alfano C. M. (2019). Cancer treatment and survivorship statistics, 2019. *CA Cancer J. Clin.* 69 363–385. 10.3322/caac.21565 31184787

[B34] Moroni E. Paladino A. Colombo G. (2015). The dynamics of drug discovery. *Curr. Top. Med. Chem.* 15 2043–2055. 10.2174/1568026615666150519102950 26156197

[B35] Muller M. Schouten R. D. De Gooijer C. J. Baas P. (2017). Pembrolizumab for the treatment of non-small cell lung cancer. *Expert Rev. Anticancer Ther.* 17 399–409. 10.1080/14737140.2017.1311791 28338376

[B36] Orellana L. (2019). Large-scale conformational changes and protein function: breaking the in silico barrier. *Front. Mol. Biosci.* 6:117. 10.3389/fmolb.2019.00117 31750315 PMC6848229

[B37] Papaleo E. Saladino G. Lambrughi M. Lindorff-Larsen K. Gervasio F. L. Nussinov R. (2016). The role of protein loops and linkers in conformational dynamics and allostery. *Chem. Rev.* 116 6391–6423. 10.1021/acs.chemrev.5b00623 26889708

[B38] Pardoll D. M. (2012). The blockade of immune checkpoints in cancer immunotherapy. *Nat. Rev. Cancer* 12 252–264. 10.1038/nrc3239 22437870 PMC4856023

[B39] Phillips J. C. Braun R. Wang W. Gumbart J. Tajkhorshid E. Villa E. (2005). Scalable molecular dynamics with NAMD. *J. Comput. Chem.* 26 1781–1802. 10.1002/jcc.20289 16222654 PMC2486339

[B40] Ribas A. Wolchok J. D. (2018). Cancer immunotherapy using checkpoint blockade. *Science* 359 1350–1355. 10.1126/science.aar4060 29567705 PMC7391259

[B41] Rittmeyer A. Barlesi F. Waterkamp D. Park K. Ciardiello F. von Pawel J. (2017). Atezolizumab versus docetaxel in patients with previously treated non-small-cell lung cancer (OAK): a phase 3, open-label, multicentre randomised controlled trial. *Lancet* 389 255–265. 10.1016/S0140-6736(16)32517-X 27979383 PMC6886121

[B42] Russo Krauss I. Ferraro G. Merlino A. (2016). Cisplatin-protein interactions: unexpected drug binding to N-Terminal amine and lysine side chains. *Inorg. Chem.* 55 7814–7816. 10.1021/acs.inorgchem.6b01234 27482735

[B43] Schmidtke P. Luque F. J. Murray J. B. Barril X. (2011). Shielded hydrogen bonds as structural determinants of binding kinetics: application in drug design. *J. Am. Chem. Soc.* 133 18903–18910. 10.1021/ja207494u 21981450

[B44] Sharma P. Allison J. P. (2015). Immune checkpoint targeting in cancer therapy: toward combination strategies with curative potential. *Cell* 161 205–214. 10.1016/j.cell.2015.03.030 25860605 PMC5905674

[B45] Sidaway P. (2017). Urological cancer: atezolizumab: an alternative to cisplatin? *Nat. Rev. Clin. Oncol.* 14:139. 10.1038/nrclinonc.2016.222 28031559

[B46] Syed Y. Y. (2017). Durvalumab: first global approval. *Drugs* 77 1369–1376. 10.1007/s40265-017-0782-5 28643244 PMC5636860

[B47] Tan S. Zhang H. Chai Y. Song H. Tong Z. Wang Q. (2017). An unexpected N-terminal loop in PD-1 dominates binding by nivolumab. *Nat. Commun.* 8:14369. 10.1038/ncomms14369 28165004 PMC5303876

[B48] Venkatasubramaniam A. Drude A. Good T. (2014). Role of N-terminal residues in Abeta interactions with integrin receptor and cell surface. *Biochim. Biophys. Acta* 1838 2568–2577. 10.1016/j.bbamem.2014.06.011 24955499

[B49] Zaidi N. Jaffee E. M. (2018). Immunotherapy transforms cancer treatment. *J. Clin. Invest.* 129 46–47. 10.1172/JCI126046 30507614 PMC6307941

[B50] Zak K. M. Kitel R. Przetocka S. Golik P. Guzik K. Musielak B. (2015). Structure of the complex of human programmed death 1, PD-1, and Its Ligand PD-L1. *Structure* 23 2341–2348. 10.1016/j.str.2015.09.010 26602187 PMC4752817

[B51] Zitvogel L. Kroemer G. (2012). Targeting PD-1/PD-L1 interactions for cancer immunotherapy. *Oncoimmunology* 1 1223–1225. 10.4161/onci.21335 23243584 PMC3518493

