# Supplementary material for: Investigating the Role of the N-Terminal Loop of PD-1 in Binding Process Between PD-1 and Nivolumab via Molecular Dynamics Simulation
Source: Front Mol Biosci. 2020 Sep 15;7:574759. doi: 10.3389/fmolb.2020.574759 (PMC7522605; doi:10.3389/fmolb.2020.574759)
Supplement: Supplementary file 1 [file Table_1.DOCX]

Supplementary Material

## Supplementary Figures





**Supplementary Figure S1.** RMSD of Complex Ⅰ in three equilibriums (Equ1, Equ2, and Equ3). Black denotes Equ1, red denotes Equ2, and blue denotes Equ3.





**Supplementary Figure S2.** RMSD of Complex Ⅱ in three equilibriums (Equ1, Equ2, and Equ3). Black denotes Equ1, red denotes Equ2, and blue denotes Equ3.





**Supplementary Figure S3.** RMSD of Complex Ⅰ-truncated in three equilibriums (Equ1, Equ2, and Equ3). Black denotes Equ1, red denotes Equ2, and blue denotes Equ3.





**Supplementary Figure S4.** RMSD of Complex Ⅱ-truncated in three equilibriums (Equ1, Equ2, and Equ3). Black denotes Equ1, red denotes Equ2, and blue denotes Equ3.





**Supplementary Figure S5.** RMSD of Complex Ⅰ-N-rotated in three equilibriums (Equ1, Equ2, and Equ3). Black denotes Equ1, red denotes Equ2, and blue denotes Equ3.





**Supplementary Figure S6.** RMSD of Complex Ⅱ-N-rotated in three equilibriums (Equ1, Equ2, and Equ3). Black denotes Equ1, red denotes Equ2, and blue denotes Equ3.





**Supplementary Figure S7.** RMSD of Complex Ⅰ-IgV-rotated in three equilibriums (Equ1, Equ2, and Equ3). Black denotes Equ1, red denotes Equ2, and blue denotes Equ3.





**Supplementary Figure S8.** RMSD of Complex Ⅱ-IgV-rotated in three equilibriums (Equ1, Equ2, and Equ3). Black denotes Equ1, red denotes Equ2, and blue denotes Equ3.
