# Supplementary material for: Investigating the Role of the N-Terminal Loop of PD-1 in Binding Process Between PD-1 and Nivolumab via Molecular Dynamics Simulation
Source: Front Mol Biosci. 2020 Sep 15;7:574759. doi: 10.3389/fmolb.2020.574759 (PMC7522605; doi:10.3389/fmolb.2020.574759)
Supplement: Supplementary file 2 [file Table_2.DOCX]

**Supplementary Table 1. Number of water molecules, Na^+^ and Cl^-^ ions added for each complex system**

| Complex | Number of water molecules | Number of Na^+^ ions | Number of Cl^-^ ions |
| --- | --- | --- | --- |
| Complex I | 35576 | 102 | 109 |
| Complex II | 34944 | 101 | 106 |
| Complex I-N-truncated | 35616 | 103 | 111 |
| Complex II-N-truncated | 35002 | 101 | 107 |
| Complex I-N-rotated | 36117 | 104 | 112 |
| Complex II-N-rotated | 34916 | 101 | 106 |
| Complex I-IgV-rotated | 38949 | 113 | 130 |
| Complex II-IgV-rotated | 38285 | 110 | 115 |
